# Consequences of Low Sleep Duration in Anthropometric and Body Composition Parameters of Chilean Preschoolers

**DOI:** 10.3390/children8010008

**Published:** 2020-12-25

**Authors:** Cristian Alvarez, Lorena Paredes-Arévalo, Isabel Obando, Marlys Leal, Yennifer Avila, Kabir P. Sadarangani, Pedro Delgado-Floody, Alicia M. Alonso-Martínez, Mikel Izquierdo

**Affiliations:** 1 Laboratory of Human Performance, Quality of Life and Wellness Research Group, Department of Physical Activity Sciences, Universidad de Los Lagos, 5290000 Osorno, Chile; cristian.alvarez@ulagos.cl; 2 Career of Nursing, Department of Health, Universidad de Los Lagos, 5290000 Osorno, Chile; lorena.paredes@ulagos.cl (L.P.-A.); isabel.obando@ulagos.cl (I.O.); marlys.leal@ulagos.cl (M.L.); 3 Nutrition and Diet Career, Department of Health, Universidad de Los Lagos, 5290000 Osorno, Chile; yennifer.avila@ulagos.cl; 4 Escuela de Kinesiología, Facultad de Salud y Odontología, Universidad Diego Portales, 8370057 Santiago, Chile; kabir.sadarangani@gmail.com; 5 Faculty of Health Sciences, School of Physiotherapy, Universidad San Sebastian, 7510157 Santiago, Chile; 6 Department of Physical Education, Sport and Recreation, Universidad de La Frontera, 01145 Temuco, Chile; pedro.delgado@ufrontera.cl; 7 Navarrabiomed, Complejo Hospitalario de Navarra (CHN)-Universidad Pública de Navarra (UPNA), IdiSNA, 31008 Pamplona, Spain; aliciamaria.alonso@unavarra.es; 8 Grupo GICAEDS, Programa de Cultura Física, Deporte y Recreación, Universidad Santo Tomás, 110311 Bogotá, Colombia; 9 CIBER of Frailty and Healthy Aging (CIBERFES), Instituto de Salud Carlos III, 28029 Madrid, Spain

**Keywords:** sleep time, obesity, adiposity, anthropometry, body composition

## Abstract

Reduced sleep duration in schoolchildren has been associated with poor health outcomes at the scholar level; however, there is little information on the effects of sleep loss on Chilean preschoolers. The aim of this study was to describe and compare cardiometabolic outcomes according with the sleep duration in Chilean preschoolers. A second aim was to identify potential risk outcomes (i.e., in odds ratios) for suffering anthropometric and body composition alterations related with participants’ sleep duration. A total of 99 (*n* = 54 boys; *n* = 45 girls) preschoolers (mean age (95% CI) 3.1 (2.7, 3.4 years), mean weight 16.1 (15.5, 16.6 kg)) were included in this study. Sleep duration was assessed using standardized questionnaires with the parents. Socio-demographic parents’ information, as well as anthropometric, nutritional, and handgrip strength outcomes in preschoolers were distributed by tertiles (T1; < 10 h, T2; 10 to < 11 h, and T3; ≥ 11 h) of sleep time. Children in the lowest tertile of sleep duration had significantly higher body mass indices (*p* = 0.036), weight-for-height Z-scores (*p* < 0.0001), waist–hip ratios (*p* = 0.041), and body fat in percent (*p* = 0.035) and kg (*p* = 0.044) compared to those in the top tertile. Low sleep duration was associated with significantly greater risks of overweight/obesity (OR 1.3 (0.9, 1.8)), low height (OR 1.2 (0.8, 1.6)), and increased BMI (OR 1.5 (0.4, 1.4)), but not with reductions in grip strength. Chilean preschoolers with low sleep duration showed higher cardiometabolic markers (anthropometric/body composition) and were more likely to be classified as obese than youth with longer sleep duration.

## 1. Introduction

Childhood obesity has become a major health challenge in the American continent, in both preschoolers and primary schoolchildren [1]. About one-third (i.e., 8.4% of preschool children (age, 2–4 years), 34.2% of school-aged children (age, 6–11 years), and 34.5% of adolescents (age, 12–19 years)) of children and adolescents report overweight/obesity [2]. In Latin American countries as Chile, there has been similarly a dramatic report in the obesity prevalence at primary school increasing from 21.5 in 2009 to 24.4% in 2017 [3]. Lack of physical activity and unhealthy eating patterns has been classically detected and studied as the main factors to increase the risk of overweight and obesity [4]. Similarly, spending children long time in TV viewing, videogames, and in general in screen exposure have a strong association with more obesity prevalence [5]. Other exposures, such as not enough sleep, high amount of stress, or parents/familial factors can be also contributors but poorly explored factors at level of preschoolers (i.e., schoolchildren ≥ 2 and ≤ 4 years old) [6]. Likewise, research on sleep duration has been inversely associated with obesity in children between 3 and 10 years of age [7]. Sleep is an important modulator of neuroendocrine function and glucose metabolism across all ages [8]. A low sleep in terms of duration and quality is associated with unhealthy dietary patterns (i.e., high fat and glucose consumption) [9] and poor glucose control (i.e., hyperglycemia and insulin resistance) [10]. Recent recommendations from the American Academy of Sleep Medicine state that children aged 6–12 years need to sleep 9–12 h per day in order to promote an optimal health status [11]. In children, robust epidemiological data have revealed that a low sleep duration has been associated with more obesity prevalence, high adiposity, and high fasting glucose levels [12]. In primary schoolchildren (i.e., children ≥ 6 years to the Chilean educational system), it was recently reported that 30 min of reduction in sleep duration was associated with (i) elevated blood pressure levels in Amerindian and European students [13,14], (ii) with lower classroom attention [15], (iii) with attention-deficit and hyperactivity disorders [16], and (iv) with lower global cognition. Other studies also shown that children of 2 years old who lose a daytime nap (i.e., in a 24 h cycle) showed more negative emotional responses [17], and reported low bone mineral content [18]. However, there is little information regarding sleep duration and their association with socio-demographic and lifestyle’s characteristics from parents in Chilean preschoolers.

Parent-child interaction also plays a very important role in either the success or failure in lifestyle modifications programs (i.e., physical activity/play; after-school physical activity participation, extra activities in general, but including screen time practices, feeding behaviors, and other more sedentary activities), affecting also the amount of sleep for pediatric populations. In this sense, as the sleep duration of preschoolers is mainly modulated by their parents, and in order to detect the early stages in which the sleep duration can be related with a more healthy/or unhealthy profile, there is a need for increasing the evidence of the sleep duration in Latin American preschoolers. In this line, considering that obesity prevalence is higher at Latin American countries, due to there being a high volume of information regarding obesity and sleep duration at the level of primary school, due to there being little information regarding the impact of the sleep duration in other more young populations, and taking into account the need for predicting obesity at the early life, the aim of this study was to describe and compare cardiometabolic outcomes according with the sleep duration in Chilean preschoolers. A second aim was to identify potentials risk outcomes (i.e., in odds ratios) for suffering anthropometric and body composition alterations related with participant’s sleep duration.

## 2. Materials and Methods

### 2.1. Design

We followed a cross-sectional design with participants (*n* = 141) parents of preschoolers (i.e., ages 2 to ≤ 4 years) of two public schools (i.e., Jardín Infantil Bosque de Colores, *n* = 144, and Jardín Infantil Semillas de Amor, *n* = 90; total population of (*n* = 234)) indexed to the governmental Chilean institution National Joint of preschools (JUNJI) of Osorno, city. All the parents/guardians signed a written informed consent after being informed in detail of the study aims and procedures by a meeting with our research team. The study was carried out under the recommendations for human studies according with the Declaration of Helsinki and was approved by the Ethical Committee of The Service of Health of Valdivia, which depends on the Ministry of Health, Chile. (ORDN°016-31012019)

**Figure 1:**
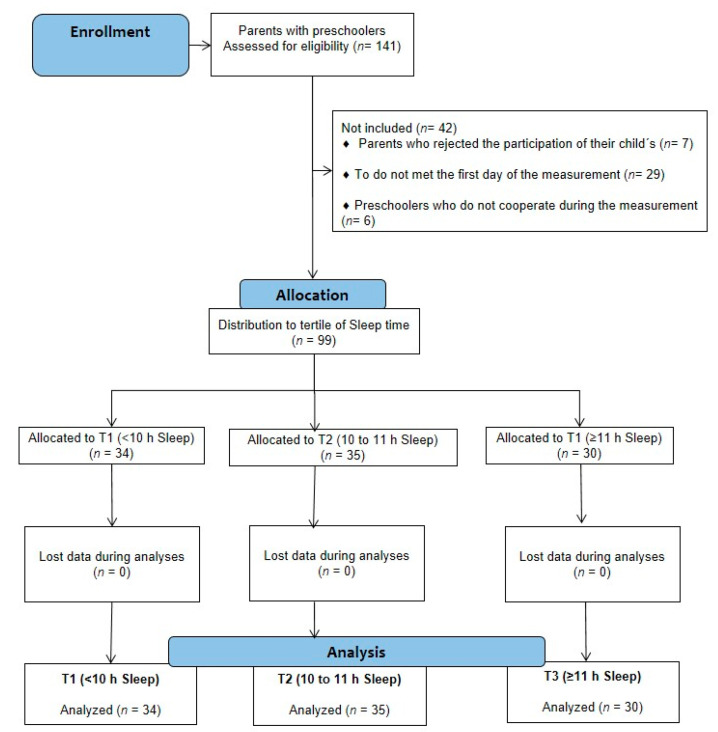
Study design. (T1: < 10 h of sleep duration; tertil 2, T2: 10 to 11 h of sleep duration; tertil 3, T3: ≥ 11 h of sleep duration)

The inclusion criteria were as follows: (a) preschoolers enrolled at one of the public schools, (b) to participate of an informative group meeting (mother/father) about the study, (c) to receive (i.e., parents) an oral personal information about the study aims (information store, and feedback for parents), (d) to participate (i.e., the preschool children) of all school activities (academic, motor plays) regularly. The exclusion criteria were as follows: (a) do not have any electronic device (i.e., participants without insulin pump, cardiovascular electronic device) that could be altered by the body composition bioelectrical impedance (BIA) analyses, (b) do not finish all the measurements, (c) in accidental dehydration (i.e., by stationary diarrheal square) reported by parents the day of the BIA body composition measurement, and (d) taking medication that induces sleep. From the enrollment stage, children were excluded (*n* = 42) due to several reasons: (1) parents who rejected the participation of their child (*n* = 7), (2) those were not present on the first day of the measurement (*n* = 29), and (3) those preschoolers who did not cooperate during the measurement (*n* = 6). Thus, according with the total population of (*n* = 234), and looking for the inclusion of 60% of the participants, we recruit a minimum sample size of (*n* = 99) preschoolers, who were categorized in three groups of tertiles. This procedure was applied using G*Power^TM^ 3.1.9.7 version, where a minimum number of (*n* = 84) participants was required for 95% of statistical power at a *p* = 0.05 of error acceptance and 95%CI. Following this, the selected preschoolers (*n* = 99) were screened by the above-mentioned outcomes, and their parents (i.e., mother or father) were given information by the questionnaire applied. The sample was distributed and analyzed by tertiles of sleep time (T1; < 10 h, age 3.0 (2.7, 3.2), T2; 10 to < 11 h, age 3.1 (2.9, 3.3), T3; ≥ 11 h of sleep time, age 3.2 (2.9, 3.4). The study design is shown in Figure 1.

### 2.2. Sleep Information from Parent’s Questionnaire

Sleeping time was measured using a questionnaire for parents/holders that included 4 items: (i) socio-demographic parents/family information (9 questions), (ii) preschooler information (4 questions), (iii) daily activities of parents with their children information (13 questions), and (iv) daily oral communication parents/children (7 questions). Similar with previous studies in primary schoolchildren from parents [19], the first stage of the study included the application of a questionnaire to the parent/holders in order to register basic socio-demographic information (mother or father, or tutor). This questionnaire also registered information regarding the family and parent’s lifestyles and habits that could potentially be correlated with markers of physical and mental health. Moreover, participant’s (i.e., preschoolers) information and extra-school activities participation between parents and preschoolers were also accounted. The application of the questionnaire was coordinated and carried out in a specific date were both school authorities intervened. Before completing the questionnaire, the parents/holders received a verbal explanation of each question included in the four items. The average time for this process to be completed was approximately 30 min. Sleep time was calculated from the difference in hours between going to bed and morning awakening (i.e., including also the nap time into a 24–4 h cycle), according to the parents’/guardians’ information.

### 2.3. Anthropometric and Body Composition Measurement

The height was measured by a stadiometer with 0.1 cm precision (Charder^TM^ SECA 213, Hamburg, Germany). The measurement of body composition included weight, body fat, muscle mass, fat free mas, fat free mass of trunk, and basal metabolic rate was developed using a digital scale (InBody 270^TM^, Biospace, Inc., Seoul, Korea) with two (20 and 100 kHz) different frequencies, tetrapolar 8-point tactile electrodes, 330 µA, with testing weight range measurement of 10 to 250 kg. This procedure was conducted in an appropriate space with previous coordination of professional staff (certified nursing and nutritionist). Thus, for approximately 30 s, the preschoolers were disposed in a standing position without any metal devices or watches, receiving stimuli (a video game play supported by our staff) using a digital screen (Tablet A 2017 Samsung Galaxy, model, SKU 535983999, Seoul, South Korea) in order to maintain the preschooler’s attention while taking the measurements. This strategy give the possibility of maintaining both hands of the preschoolers in contact with the hand section of the BIA equipment during the 30 s of time required without movement for data evaluation and recording. These procedures increased the time and the efficiently of the measurements, allowing children to stand still. With the weight and height, we calculated the body mass index (BMI) by dividing the weight by the square of their height in meters (kg/m^2^). All participants data of BMI was classified according to the cut-off points for age and sex proposed by the World Health Organization (2007) [20] and the International Obesity Task Force (2012) [21]. This procedure is regularly applied by the Chilean public health government to children nutritional state classification [22]. Anthropometric and body composition measurements were carried out between 9 and 13 h in the morning.

### 2.4. Muscle Strength Measurement

Each participant was evaluated in their muscle strength through the handgrip muscle strength in the dominant (HGSd), and nondominant (HGSnd) arm. This procedure was executed into an adapted room into the school, in the morning (i.e., from 9 to 12 h) in a sit position, with the elbow joint in 90° and using an adjustable grip (Jamar, PLUS+MR, Sammons Preston, Patterson Medical, Bolingbrook, IL), similar to previous studies carried out in schoolchildren [23]. Participants were continuously instructed to gradually tighten their grip for at least 5 s of maximal effort in each hand while receiving standardized verbal motivation. After this procedure, the data were translated immediately to an Excel sheet for future analyses. The parents’ socio-demographic and characteristics of the schoolchildren are shown in Table 1.

### 2.5. Statistical Analyses

Participants were grouped into tertiles (T1; < 10 h, T2; 10–11 h, and T3; > 11 h) of sleeping time. The first tertile (T1, < 10 h) was used as the reference in most cases, and multiple comparisons were made across T1 and T3. All outcomes were tested for normality by the Kolmogorov–Smirnoff method. The descriptive characteristics of the cohort are presented as means ± (95% CI) to continuous, or as frequency (*n* = ) plus percentage (%) to categorical outcomes, respectively (Table 1). With the weight and height outcomes, we obtained the weight/height ratio outcome. Form here, this was transformed to a z score and we obtained the z-weight/height outcome. The differences between means of tertiles by each outcome were analyzed with the Kruskall–Wallis test. The reference tertile (Ref) in anthropometric and body composition outcomes was estimated as those participants whose adherence to 12-h of sleep duration (i.e., over a 24-h cycle), and it is shown as Ref in each figure. Associations between the sleep patterns by tertiles of sleeping time were analyzed using the General Linear Model (with age, BMI, and gender as covariates), obtaining the beta coefficients and their 95% CI. Additionally, the *p*-trend was calculated to test the worsening of the outcomes across the tertiles. Eventually, the odds ratios (OR) and 95% confidence intervals (CI) for the suffering of more (a) anthropometric; overweight/obesity prevalence, higher weight, low height, higher weight/height ratio, higher z-score of weight/height ratio, higher BMI, and higher waist/hip ratio (i.e., a value superior than the mean of the gender sample), and (b) body composition; increased body fat (expressed in kg and %), a decreased fat-free mass of the trunk (expressed in kg and %), increased waist/hip ratio, decreased muscle mass, and a decreased basal metabolic rate (i.e., a value superior than the mean of the gender sample). The Reference bar (Ref) to OR figures was estimated from sleep time ≥ 11 h (over a 24-h cycle). All statistical analyses were conducted using SPSS v23 software (SPSS Inc., Chicago, IL), with α set at 0.05.

## 3. Results

### 3.1. Anthropometric Outcomes by Tertiles of Sleep Duration

**Figure 2:**
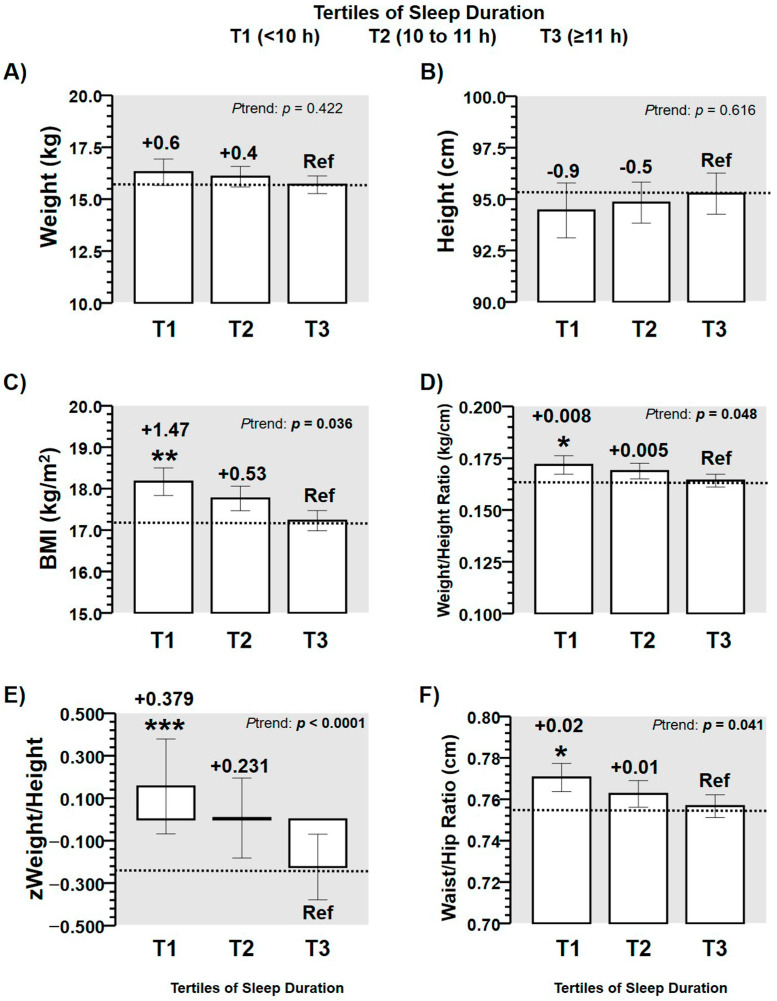
Characteristics of anthropometric outcomes in a sample of Chilean preschoolers (*n* = 99) by tertiles of sleep duration, described as Table 1. (T1: < 10 h of sleep duration; tertile 2, T2: 10 to 11 h of sleep duration; tertile 3, T3: ≥ 11 h of sleep duration, that was used as the Reference (Ref) category). (BMI) Body mass index in kg/m^2^. Panel (**A**) describe weight, (**B**) height, (**C**) BMI, (**D**) weight/height Ratio, (**E**) z score of weight/height Ratio, and (**F**) waist/hip Ratio across tertiles of sleep duration. (*) Denotes significant differences between tertile of origin vs. tertile Reference at *p* < 0.05. (**) Denotes significant differences between tertile of origin vs. tertile Reference at *p* < 0.01. (***) Denotes significant differences between tertile of origin vs. tertile Reference at *p* < 0.0001.

In anthropometric outcomes, no significant differences were observed in weight and height comparing tertiles of sleep duration (Figure 2A,B). However, a significant higher values in *P*trend (*p* < 0.05) were found across tertiles of sleep duration in outcomes BMI (T2 + 0.4, T1 +0.6 kg/m^2^ than T3 Reference (Ref), *p* = 0.036) (Figure 2C), weight/height ratio (T2 + 0.005, T1 + 0.008 kg/cm than T3 Ref, *p* = 0.048) (Figure 2D), z-weight/height (T2 + 0.231, T1 + 0.379 than T3 Ref, *p* < 0.0001) (Figure 2E), and waist-to-hip ratio (T2 + 0.01, T1 + 0.02 kg/m^2^ than T3 Ref, *p* = 0.041) (Figure 2F).

### 3.2. Body Composition Outcomes by Tertiles of Sleep Duration

**Figure 3:**
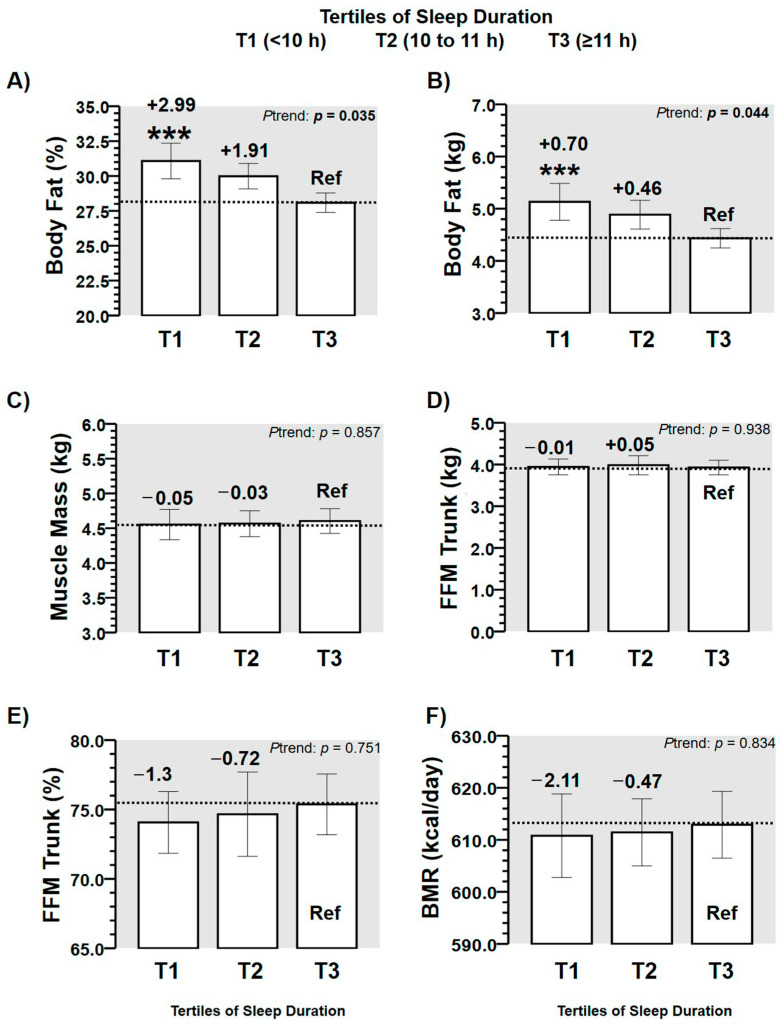
Characteristics of body composition outcomes in a sample of preschoolers (*n* = 99) by tertiles of sleep duration, described as tertile 1 (T1: < 10 h of sleep time; tertile 2, T2: 10 to 11 h of sleep duration; tertile 3, T3: ≥ 11 h of sleep duration, that was used as the Reference category). (FFM) Fat free mass of trunk) Fat-free mass of trunk in kg or %, (BMR) Basal metabolic rate in kcal/day. Panel (**A**) describe body fat in percentage, (**B**) body fat in kilograms, (**C**) muscle mass, (**D**) fat free mass of trunk in kilograms, (**E**) fat free mass of trunk in percentage, and (**F**) basal metabolic rate, across tertiles of sleep duration. (***) Denotes significant differences between tertile of origin vs. tertile Reference at *p* < 0.0001.

In body composition outcomes, body fat percentage showed significant differences across tertiles of sleep duration by the *p*-trend (T2 + 1.91, T2 + 2.99 % than T3 Ref, *p* = 0.035) (Figure 3A). Similarly body fat in kg also showed significant differences across tertiles of sleep duration by the *p*-trend (T2 + 1.46, T2 + 0.70 kg than T3 Ref, *p* = 0.044) (Figure 3B). However, in the other body composition outcomes (i.e., muscle mass, fat-free mass of the trunk (in kg and %), and basal metabolic rate), no significant differences between tertiles of sleep duration were found (Figure 3C–F).

### 3.3. Odds Ratios for Suffering More Altered Anthropometric Markers of Adiposity

**Figure 4:**
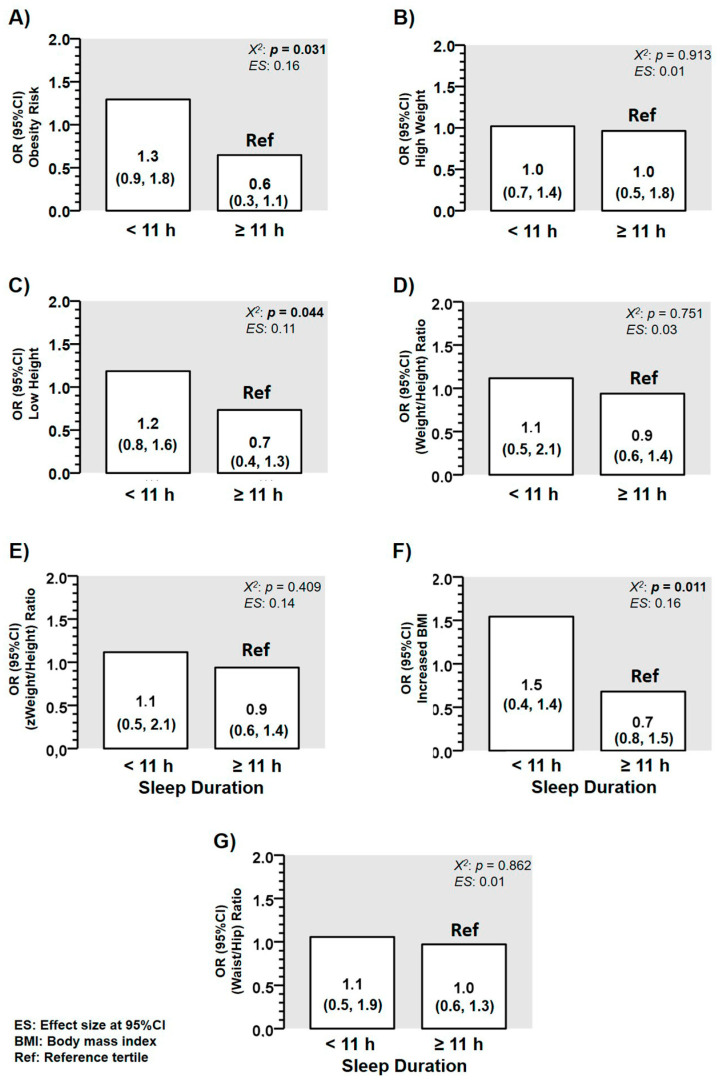
Characteristics of different anthropometric outcomes in a sample of Chilean preschoolers (*n* = 99) according with their odds ratio (OR) for suffering or not the condition of (**A**) OR for suffering from higher “overweight/obesity” prevalence, (**B**) OR for suffering from “excess from weight”, (**C**) OR for suffering from “low height”, (**D**) OR for suffering from “elevated weight/height ratio”, (**E**) OR for suffering from “elevated z score weight/height ratio”, (**F**) OR for suffering from “elevated waits-to-hip ratio”, and (**G**) OR for suffering from “elevated waist/hip ratio. (ES) Denotes Cohen d effect size.

Obesity odds ratios (ORs) for preschoolers who reported low sleep duration was higher comparing preschoolers with high sleep duration < 11 h; OR 1.3 (0.9, 1.8) vs. those with ≥ 11 h; OR 0.6 (0.3, 1.1) Ref, *p* = 0.031) (Figure 4A). There was a higher risk for suffering from “low height” comparing preschoolers with low (< 11 h; OR 1.2 (0.8, 1.6) vs. those peers with ≥ 11 h; OR 0.7 (0.4, 1.3) Ref, *p* = 0.044) of sleep duration (Figure 4C). There was a higher risk for suffering from “increased BMI” comparing preschoolers with low sleep time (< 11 h; OR 1.5 (0.4, 1.4) vs. those peers with ≥ 11 h; OR 0.7 (0.8, 1.5) Ref, *p* = 0.011) of sleep duration (Figure 4F). In contrast, there were no differences among categories of ORs for suffering of “high weight” (Figure 4B), “higher weight/height ratio” (Figure 4D), “higher z-weight/height ratio” (Figure 4E), and “higher waist/hip ratio” (Figure 4G).

### 3.4. Odds Ratios for Suffering from More Altered Body Composition Markers of Adiposity

**Figure 5:**
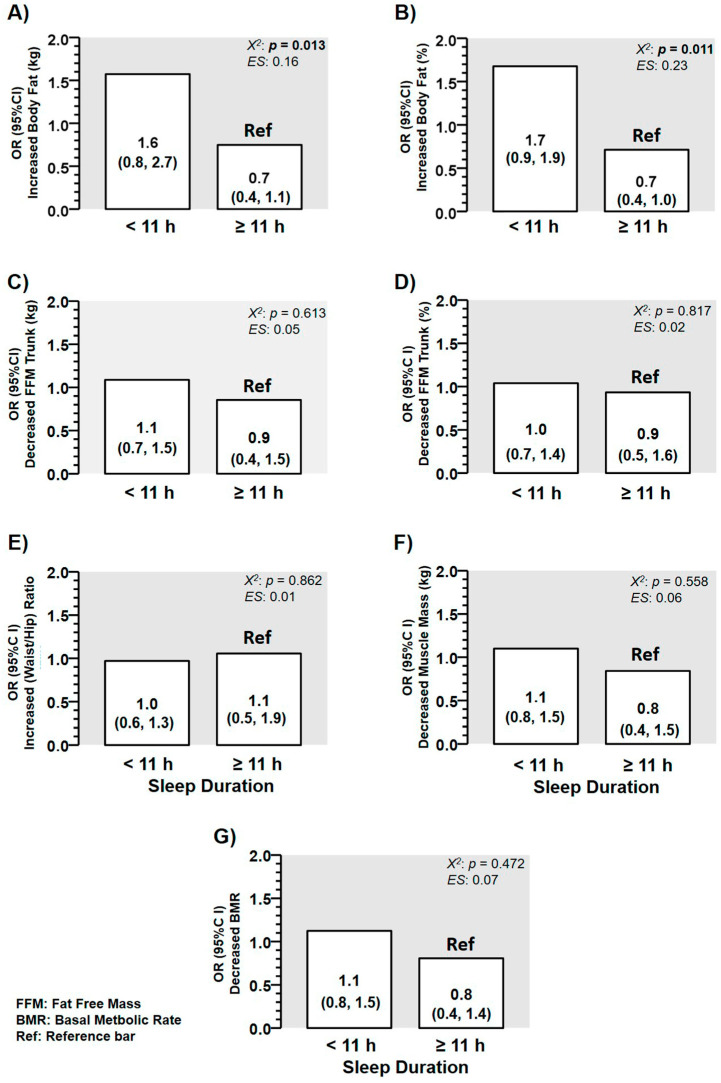
Characteristics of different body composition outcomes in a sample of Chilean preschoolers (*n* = 99) according with their odds ratio (OR) for suffering or not the condition of (**A**) OR for suffering from increased “body fat in kg”, (**B**) OR for suffering from increased “body fat in percentage”, (**C**) OR for suffering from “decreased fat free mass in the trunk in kg”, (**D**) OR for suffering from “decreased fat-free mass in the trunk in percentage”, (**E**) OR for suffering from “increased waist to height ratio”, (**F**) OR for suffering from decreased muscle mass”, (**G**) OR for suffering from “decreased basal metabolic rate at rest”. (ES) Denotes Cohen d effect size.

There was a higher risk for suffering from “increased body fat” in kg comparing preschoolers with low (<11 h; OR 1.6 (0.8, 2.7) vs. those with ≥11 h, OR 0.7 (0.4, 1.1) Ref, *p* = 0.013) of sleep duration (Figure 5A). Similarly, there was a higher risk for suffering from “increased body fat” in % comparing preschoolers with low (< 11 h; OR 1.7 (0.9, 1.9) vs. those with ≥ 11 h; OR 0.7 (0.4, 1.0) Ref, *p* = 0.011) of sleep duration (Figure 5B). By contrast, there were no differences among categories of OR comparing “low” vs. “high” sleep duration in categories of ORs for “decreasing fat free mass of trunk in kg” (Figure 5C), “decreasing fat free mass trunk in %” (Figure 5D), to “increase waist/hip ratio” (Figure 5E), for “decreasing muscle mass” (Figure 5F), and to “decrease basal metabolic rate” (Figure 5G).

### 3.5. Association of the Sleep Time with Z-Weight/Height Ratio and Body Fat Percentage

Table 2 shows four models (model 1; sleep time, model 2; sleep time + age; model 3 sleep time + age + gender, and model 4; sleep time + age + gender + handgrip strength) based off sleep time in combination with other outcomes adjusted for predicting changes in z-weight/height and body fat percentage. The changes in z-weight/height were significantly predicted from the four models of sleep time adjusted by sleep time + age (model 2 (20.3%, *p* < 0.0001), age + gender (model 3; 22.0%, *p* < 0.0001), and age + gender + handgrip strength (model 4; 28.8%, *p* < 0.0001). Similarly, considering the four models, the changes in body fat percentage were significantly predicted from sleep time adjusted by sleep time alone (model 1; 2.8%, *p* < 0.0001), + age (model 2; 20.3%, *p* < 0.0001), sleep time + age (model 2; 2.9%, *p* < 0.0001), age + gender (model 3; 7.2%, *p* < 0.0001), and by age + gender + handgrip strength (model 4; 8.3%, *p* < 0.0001).

## 4. Discussion

The aim of this study was to describe and compare cardiometabolic outcomes according with the sleep duration in Chilean preschoolers. A second aim was to identify potentials risk outcomes (i.e., in odds ratios) for suffering from anthropometric and body composition alterations related with participant’s sleep duration. The main findings of the present study were that Chilean preschoolers with low sleep duration showed (i) greater body weight, higher body fatness, increased risk of obesity, and (ii) higher risk for suffering of more obesity, low height, and body fat than peers who had more sleep duration. Thus, Chilean preschoolers with low sleep duration showed higher cardiometabolic markers (anthropometric/body composition) and were more likely to be classified as obese as youth with longer sleep duration. These findings support that in preschoolers, there is a similar pattern of cardiometabolic risk when these show a low sleep duration than those previous findings developed in schoolchildren.

Although the association of time of sleep duration with adiposity markers has been explored in American schoolchildren aged ≥ 6 years [6,10], adolescents [24,25], as well as in adults [26], there is little information on Latin American preschoolers. Among previous evidence, Carson et al. [27] showed in 3 to 4-year-old preschoolers that sleep duration was not significantly associated with BMI and waist circumference in Canadian participants (*n* = 552). By contrast, another study using high technology equipment for body composition (i.e., Dual-energy X-ray Absorptiometry (DXA) developed in preschoolers of 4 years) reported that a low sleep duration was significantly correlated with higher BMI in (kg/m^2^), higher fat mass in (kg), and greater fat-free mass index (kg) [28]. On the other hand, a recent study reported the sleep duration in children between 2 and 9 years old of eight European countries as follows; 9.5 h in Estonia and Italy, of ≈10.5 to 11 h in Sweden, Germany, and Belgium of ≈10.5 to 11 h [1]. In comparison with these data of sleep duration, our preschooler sample had by contrast, a lower range of age (i.e., 2 to < 4 years), where the sleep patterns reported from parents of our participants (T1; 9.0 (8.8, 9.2) h, T2; 10.1 (10.0, 10.9) h, T3 11.2 (11.1, 11.6) h) can have more confidence than these European data considering the wide biological maturation among their participants. In this line, we found that the z-weight/height was also higher in those preschoolers who showed low sleep duration (Figure 2E). It has been previously reported that the z-weight/height index has similar sensitivity as cardiometabolic risk factor related with changes in sleep duration than other more specific outcomes such as body fat or physical inactivity regularly associated with health outcomes [29]. For example, previous studies have reported that one hour more of sleep duration in children has been associated with −0.2 lower body mass index, with −0.03 kg/m^2^ lower of body fat, with −2.9% lower of homeostasis model assessment of insulin resistance, and with −0.24% lower fasting glucose [30]. In addition, in students 8 to 11 years old, higher levels of body fat have been also reported as an independent risk factor for cardiometabolic diseases related with the sleep duration and physical activity [31]. Likewise, as sleep duration plays a pivotal role in several well-being outcomes of schoolchildren, this study reveals that anthropometric and body composition outcomes are altered in order to describe poor health in Latin American Chilean preschoolers. This result is in agreement with previous studies showing that sleep duration was a better predictor of body fat percentage (R^2^ = 0.042, β = −0.052) [32]. In the present study, when preschoolers sleep <10 h (Tertile 1), participants increase the body fat percentage by +2.9% in comparison with peers who report ≥11 h of sleep duration (Tertile 3 Ref) (Figure 3A).

In the first 2 months of life, it has been previously shown that infants with a low sleep duration are those that suffer from more detrimental health-related consequences until 6 years old [33]. Thus, one may speculate about the potential role of parents/family in the sleep duration of children [34], since in the first stages of life, parents are the main ones responsible (i.e., at least environmentally) to regulate the sleep patterns (i.e., sleep duration, quality, etc.); however, due to biological or other health reasons, the sleep patterns can be also conditioned in children. Unfortunately, in the stages of these years (from 2–4 years old), it is not usual for the public education to monitor the sleep duration together for the purpose of avoiding future cardiometabolic diseases associated with the low sleep, due to there being a need for several mechanisms whose implementation would be a challenge, as well as the appropriate coordination with health institutions. The results of the present study are also in agreement with the association of sleep duration with obesity, fasting glucose [12], blood pressure [13], lower classroom attention [15], and attention-deficit/hyperactivity disorder diagnosis [16], with negative emotional responses [17]. Thus, in our sample examined (i.e., aged 3.1 (2.7, 3.4) years), we reported that those participants who sleep < 11 h showed an increased risk for suffering of overweight/obesity and “low height” (Figure 4C). Furthermore, the sleep duration predicted two major outcomes used for children who were diagnosed as overweight at young ages such as weight/height in z score from R^2^ 20.3 to 28.8%, as well as the body fat percentage from 2.8 to 8.3% (Table 2). These results corroborate the information of significantly higher z-weight/height + 0.379 in T1 in low sleep duration (< 10 h) in comparison with Ref T3, in which the sleep duration was longer (≥ 11 h) (Figure 2E), since the tendency for accumulating more body fat kg in T1 + 0.70 kg in those preschoolers participants with low sleep duration (Figure 3B) was similar. Sleep duration information is a topic that currently has been part of relevant American studies promoting an integral healthy growing in children of school age, and it has been added to other relevant topics and needs such as physical activity and screen time recommendations for schoolchildren 9 to 11 years [35]. Thus, although the American Academy of Sleep Medicine state that children aged 6–12 years need to sleep 9–12 h per day for children ≤ 12 years, and other guidelines recommend similar dosage of sleep for ≤6 years children, in Chile, there are unknown sleep patterns for this Latin-American cohort. For example, other more developed countries have reported relevant degrees of information about sleep guidelines recommendations adherence as Canada (83.9% of rate adherence), which also include screen time and daily physical activity [35]. Thus, considering that at the primary school, the sleep duration has a relevant association with adiposity and health-related markers, there is relevant interest in the addition of more evidence at early school ages such as preschool.

As potential limitations, (i) we did not include genetic markers of obesity and did not screen potential associations of familial (i.e., parents) obesity inheritance of participants, (ii) we did not use electronic objective measurements of sleep duration such as accelerometers but applied commonly used questionnaires to parents/holders, who gave us directly the information required, (iii) we did not include weekends sleep duration, and we could have overestimated the sleep duration reported during the week, and (iv) although the BIA has not been previously validated for body composition use in children younger than 3 years, the functionality of the equipment is guaranteed for individuals greater than 10 kg. In our sample, all participants were greater than 10 kg (mean weight 16.1 kg, range (11.7–25.4 kg)). Future research can be done to support the validity of this tool in preschool age children. Some strengths were that we measured directly by electronic bio-impedance (BIA) the body composition of participant’s schoolchildren and included a strength fitness outcomes as the handgrip strength in the participants for future comparisons.

## 5. Conclusions

Chilean preschoolers with low sleep duration showed higher cardiometabolic markers (anthropometric/body composition) and were more likely to be classified as obese than youth with longer sleep duration.

## Figures and Tables

**Table 1:** Parents’ socio-demographic and health-related preschooler’s characteristics of the sample.

Outcomes	Tertiles of Sleep Time			
	T1 (< 10 h) Mean (95% CI)	T2 (10 to 11 h) Mean (95% CI)	T3 (≥ 11 h) Mean (95% CI)	*p*-Trend
(*n* = )	34	35	30	
Parent´s information				
Mother Age (years)	32.0 (27.3, 36.6)	32.5 (29.8, 35.1)	32.1 (29.8, 34.5)	*p* = 0.955
Father Age (years)	33.1 (27.8, 38.4)	36.3 (32.4, 40.3)	34.3 (31.8, 36.9)	*p* = 0.797
Familiar mean income (Chilean pesos $)				
<350,000	9 (40.9%)	8 (36.4%)	5 (22.7%)	*p =* 0.719
350,000–500,000	1 (5.6%)	9 (50.0%)	8 (44.4%)	
500,000–1,000,000	3 (14.3%)	9 (42.9%)	9 (42.9%)	
>1,000,000	6 (31.6%)	7 (36.8%)	6 (31.6%)	
Mother Education				
Primary/secondary	10 (52.6%)	12 (34.3%)	10 (33.3%)	*p* = 0.214
Technical/University	8 (42.1%)	21 (60%)	20 (66.7%)	
Father Education				
Primary/secondary	10 (52.6%)	12 (34.3%)	12 (40.0%)	*p* = 0.090
Technical/University	6 (31.6%)	18 (51.4%)	18 (60%)	
Prevalence of active’s play with children at home				
Yes, *n* = / (%)	18 (94.7%)	31 (88.6%)	25 (83.3%)	*p* = 0.454
No, *n* = / (%)	1 (5.3%)	4 (11.4%)	5 (16.7%)	
Prevalence for Mother´s or Father’s play more with children at home				
Mother, *n* = / (%)	15 (78.9%)	24 (68.6%)	26 (86.7%)	*p* = 0.244
Father, *n* = / (%)	4 (21.1%)	11 (31.4%	4 (13.3%)	
Preschoolers information				
Sex, Girls	11 (57.9%)	22 (62.9%)	18 (60.0%)	
Age (years)	3.0 (2.7, 3.2)	3.1 (2.9, 3.3)	3.2 (2.9, 3.4)	*p* = 0.292
Anthropometric				
Body Fat, kg/HGS, kg Ratio	0.561 (4.059, 0.662)	0.555 (0.430, 0.670)	0.601 (0.498, 0.701)	*p* = 0.590
Body Fat, %/HGS, kg Ratio	0.713 (0.576, 0.850)	0.652 (0.535, 0.769)	0.664 (0.558, 0.770)	*p* = 0.646
Muscle Mass, kg/Body Fat, kg Ratio	0.944 (0.807, 1.081)	0.989 (0.833, 1.095)	1.061 (0.982, 1.140)	*p* = 0.132
Nutritional status				
Normal weight, *n* = / (%)	5 (26.3%)	13 (37.1%)	15 (50.0%)	*p* = 0.716
Overweight, *n* = / (%)	7 (37.8%)	12 (34.3%)	12 (40.0%)	
Obese, *n* = / (%)	7 (37.8%)	10 (28.6%)	3 (10.0%)	
Muscle strength, kg				
HGS-ra (kg)	2.9 (2.1, 3.6)	2.6 (2.1, 3.1)	3.0 (2.3, 3.6)	*p* = 0.696
HGS-la (kg)	2.8 (2.1, 3.4)	2.5 (2.0, 3.0)	2.6 (2.3, 2.9)	*p* = 0.760
Mean HGS (kg)	2.8 (2.2, 3.4)	2.5 (2.0, 3.0)	2.6 (2.1, 3.2)	*p* = 0.789

Data presented as mean for continuous variables or frequency and percentage of cases for categorical variables, with their corresponding 95% CI. (HGS) Handgrip muscle strength, (HGS-ra) Handgrip muscle strength of right arm, (HGS-la) Handgrip muscle strength of left arm.

**Table 2:** Association between sleep duration with different anthropometric (z-weight/height) and body composition (body fat percentage) outcomes in Latin American Chilean preschoolers.

	Z Weight/Height Ratio			Body Fat %		
	Predicted % by R^2^	β (95% CI)	*p*-Value	Predicted % by R^2^	β (95% CI)	*p*-Value
Model 1, Sleep time	0.008 (0.8%)	−0.092 (−0.345, 0.141)	*p* = 0.406	0.028 (2.8%)	−0.168 (−2.157, 0.273)	*p* < 0.0001
Model 2, Sleep time, age	0.203 (20.3%)	−0.154 (−0.393, 0.051)	*p* < 0.0001	0.029 (2.9%)	−0.164 (−2.152, 0.317)	*p* < 0.0001
Model 3, Sleep time, age, gender	0.220 (22.0%)	−0.154 (−0.392, 0.049)	*p* < 0.0001	0.072 (7.2%)	−0.163 (−2.129, 0.301)	*p* < 0.0001
Model 4, Sleep time, age, gender, handgrip strength	0.288 (28.8%)	−0.134 (−0.362, 0.064)	*p* < 0.0001	0.083 (8.3%)	−0.155 (−2.088, 0.350)	*p* < 0.0001

(Model 1) unadjusted “Sleep time” model. (Model 2) “Sleep time” model adjusted for “age”. (Model 3) “Sleep time” model adjusted for age and gender. (Model 4) “Sleep time” model adjusted for age, gender and handgrip strength. Bold values denotes significant.

## Data Availability

The data presented in this study are available on reasonable request from the corresponding author.
